# Antithrombotic therapy and cavernoma bleeding: does vascular border zone localization matter?

**DOI:** 10.3389/fsurg.2025.1577820

**Published:** 2025-04-30

**Authors:** Alexandru Guranda, Tim Wende, Martin Vychopen, Erdem Güresir, Ulf Nestler

**Affiliations:** Department of Neurosurgery, University Hospital Leipzig, Leipzig, Germany

**Keywords:** cavernous malformation, cavernoma, bleeding risk, vascular border zone, antithrombotic treatment

## Abstract

**Objective:**

Hemorrhagic events in cavernous malformations (CCMs) are linked to significant morbidity and mortality. Identifying factors contributing to bleeding is crucial for effective clinical and surgical management.

**Methods:**

This retrospective, observational, single-center study assessed potential and known risk factors for bleeding in patients with cerebral cavernous malformations. We evaluated age, gender, smoking habits, arterial hypertension, antithrombotic medication, diabetes mellitus, cavernoma shape, size changes observed in follow-up MRIs, the occurrence of epileptic seizures, and the localization of the lesion in regard of cerebral vascular territories.

**Results:**

We identified 143 patients (43.4% male, 45.5% with hemorrhagic events, and 19.6% with epileptic seizures). Antithrombotic medication was associated with a lower frequency of hemorrhage (*p* = 0.012). Arterial hypertension, diabetes mellitus, smoking habits and localization in the border zone of a vascular territory or seizures were not significantly associated with bleeding events. An increased rate of hemorrhagic events was found in cavernomas with irregular MRI shape (*p* = 0.001), or with changes in size during follow-up (*p* = 0.012). Multivariate analysis confirmed that antithrombotic therapy was associated with a reduced risk of hemorrhage (OR: 0.151, 95% CI: 0.041–0.552, *p* = 0.004), while male gender (OR: 2.114, 95% CI: 1.047–4.269, *p* = 0.037), irregular cavernoma shape (*p* = 0.001), and cavernoma growth (*p* = 0.002) were independently associated with a higher bleeding risk. Cavernoma localisation in the border zone between median and posterior arterial territories was associated with a significant lower rate of bleeding events compared to localisation in other watershed areas.

**Conclusion:**

We observed a reduced percentage of bleeding in cavernoma patients utilizing antithrombotic agents, compared to patients without antithrombotic medication. Factors such as smoking habits, irregular cavernoma shape on MRI, and changes in size during follow-up were associated with a higher frequency of bleeding events.

## 1 Introduction

Cerebral cavernous malformations (CCMs) are rare vascular anomalies occurring in approximately 0.4%–0.5% of the population, with an estimated annual incidence of hemorrhage about 0.7%–1% (1). Given their tendency to remain asymptomatic, many CCMs are discovered incidentally, approximately in 40.6% of cases (2).

Neurological symptoms depend on the occurrence of a bleeding event and from the localization of the lesion in the central nervous system (3). Cavernoma-related epilepsy has been found in about 40%–70% percent of patients (4, 5). Lesions in the temporal lobe are particularly epileptogenic and often require surgical intervention (6). Another frequent symptom leading to MR imaging and detection of CCMs is cephalgia. For incidentally detected and asymptomatic cavernomas, a conservative treatment approach, such as a watch-and-wait strategy, may be an appropriate option, as supported by prior studies (7, 8). However, this approach requires careful consideration, as there remains a risk of hemorrhage, which can lead to serious or even life-threatening consequences (9, 10). Therefore, accurate stratification of bleeding risk is essential to identify patients who are more likely to benefit from surgical intervention. This approach helps ensure that the risks associated with surgery, such as potential neurological deficits, are justified by a significant reduction in the probability of a major hemorrhagic event. Developing and refining predictive models for individual bleeding risk is crucial in supporting therapeutic decision-making and improving patient outcomes.

The pathophysiological mechanisms underlying cavernoma hemorrhage are still not completely understood. Endothelial shear stress, loss of cell-junctions and alteration of the coagulation cascade by surface proteins have been discussed (11). Experimental data points to a correlation of hypoxia and formation of thrombosis in cavernoma tissue, the subsequent venous congestion being involved in rupture of the vessel (12, 13). However, while these mechanisms are increasingly understood, their practical implications for patient management remain uncertain.

We hypothesized, that in arterial vascular border zones, the experimentally described mechanisms such as slow blood flow and relative hypoxia are more prevalent. This might then lead to a higher rate of cavernoma incidence and detection of hemorrhage in these areas. To test this hypothesis, in addition to the previously described risk factors for cavernoma hemorrhage, MR-imaging was assessed concerning the localization of cavernoma in arterial vascular border zones and the respective rates of hemorrhage.

## 2 Methods

### 2.1 Study design, setting, and participants

We conducted a single-center, retrospective cohort analysis using data from a database of pediatric and adult patients diagnosed with cavernous malformations at the Department of Neurosurgery, University Hospital of Leipzig, Germany, between 2000 and 2023. The study included patients with cranial and spinal lesions, encompassing both previously treated, or incidentally discovered cavernous malformations, as well as symptomatic and asymptomatic individuals.

All patients had undergone high-resolution MRI or CT scans. MRI protocols included T1-, T2-weighted imaging and susceptibility-weighted imaging (SWI). Patient data was extracted from clinical medical records. In three cases, diagnosis was based on CT imaging alone due to emergency conditions or contraindications for MRI. All CT-based diagnoses were confirmed within an interdisciplinary team and followed recognized imaging criteria for cavernoma.

### 2.2 Terms and definitions

Hemorrhage at the time of diagnosis was assessed based on radiologic evidence of recent blood products within or surrounding the cavernous malformation. All patients with suspicion of cavernoma hemorrhage received an emergency scan (MRI/CT). We classified hemorrhage according to the Angioma Alliance reporting standards, ensuring consistency with international guidelines. A symptomatic hemorrhage was defined as radiologically confirmed acute hemorrhage (*via* CT or MRI) accompanied by acute or subacute clinical symptoms such as focal neurological deficits, seizures, or severe headache (14).

Lesion size was measured using axial, sagittal, and coronal MRI images (T2 sequences, T1 sequences, and SWI) or CT scans, based on the greatest diameter observed in the first imaging at diagnosis, or as documented in outpatient reports where the diameter was already specified. Only the visible cavernoma was measured; associated hematoma, if present, was not included in the measurement. Changes in size during follow-up were defined as an increase of ≥1 mm in any dimension, measured using the same imaging modality. Cavernomas were categorized based on their morphology as cyclic (rounded or symmetrical), polycyclic (irregular or lobulated), or unknown (indeterminate due to imaging limitations).

**Figure 1:**
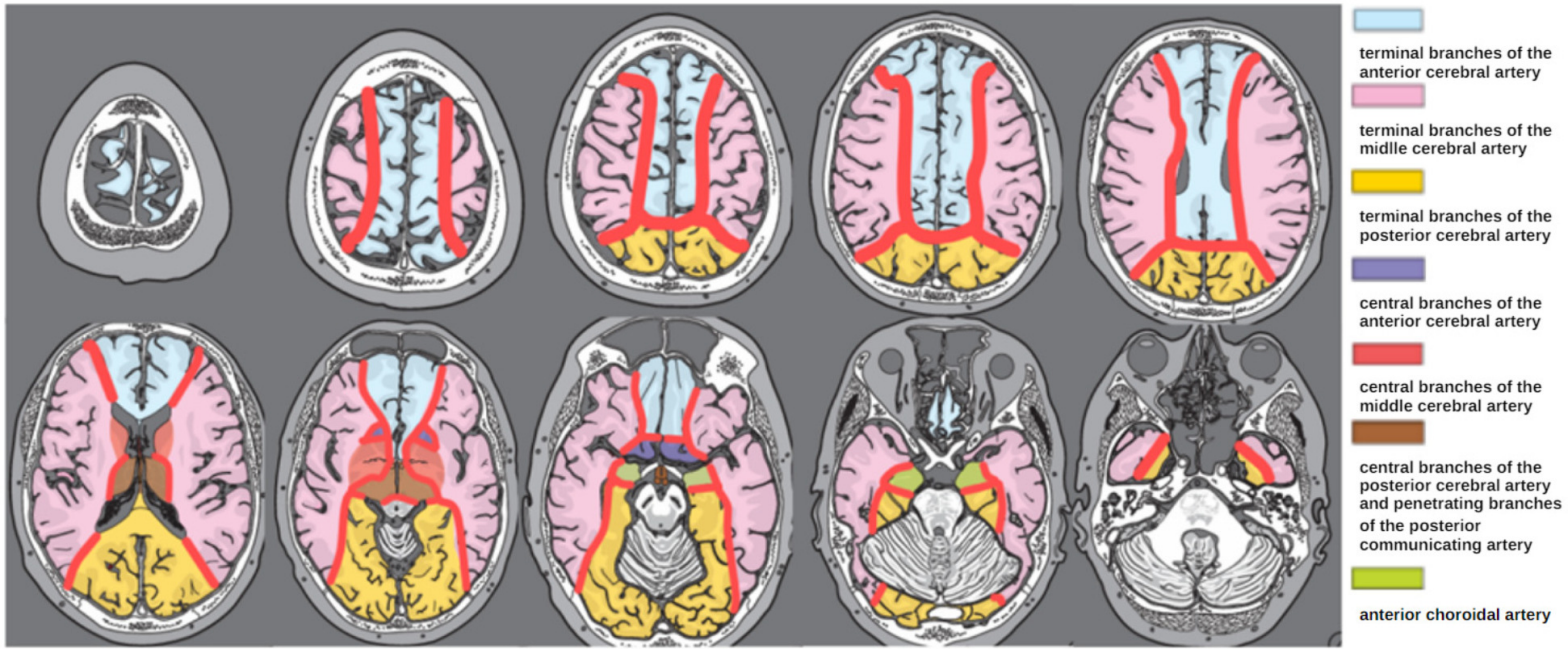
Schematic representation of the areas of arterial vascular border zones (red) employed for MRI-based cavernoma localization in axial views. Modified from Lanfermann et al. (15).

The vascular border zone was defined as the area located at the junction of major cerebral arterial territories, as the so-called watershed areas. In the context of cavernous malformations, these zones represent regions where blood flow is slowing down in the capillary bed, potentially increasing the risk for relative hypoxia or thrombotic events (12). Due to reduced oxygen transfer in these areas, the risk of relative hypoxia is further exacerbated, which may contribute to the pathophysiology of cavernoma formation or hemorrhage. The arterial territories and border zones were defined according to a clinical neuroanatomic atlas (15), MRI localization was assessed in axial, sagittal and coronal views by two examiners in consensus (AG, UN, Figure 1).

For detailed localization of cavernomas within vascular border zones, lesions were classified according to arterial supply territories into the following subtypes: ACA/ACM (border zone between the anterior cerebral artery and middle cerebral artery), ACM/ACP (middle cerebral artery and posterior cerebral artery), ACP/AB (posterior cerebral artery and basilar artery), and ACA/ACP (anterior and posterior cerebral artery) (Figure 1).

### 2.3 Baseline characteristics and management data

We investigated the known risk factors for bleeding in cerebral cavernous malformations from the archived records and radiographic images, including age, gender, smoking status, hypertension, antithrombotic medication usage, diabetes mellitus, cavernoma morphology, changes in size observed in follow-up MRIs and occurrence of epileptic seizures.

Arterial hypertension was documented when recorded as a clinical diagnosis, or if patients were on antihypertensive medication. Data on antithrombotic medication, smoking, seizure occurrence and diabetes mellitus were extracted from available inpatient and outpatient records.

### 2.4 Study endpoint

The statistical analyses were performed using IBM SPSS Statistics (Version 29; IBM Corp., Armonk, NY, USA). Descriptive statistics were generated for all baseline characteristics. Continuous variables were expressed as means ± standard deviation (SD) or medians, depending on data distribution, while categorical variables were reported as frequencies and percentages.

For univariate analysis, Fisher's exact tests were used for categorical variables. Binary logistic regression models were built to assess the independent effects of risk factors on hemorrhage, with odds ratios (ORs) and 95% confidence intervals (CIs) reported for all variables. Multicollinearity was assessed using the variance inflation factor (VIF).

All *p*-values were two-tailed, and a *p*-value <0.05 was considered to be statistically significant.

## 3 Results

### 3.1 Baseline characteristics

In total, 143 patients were included, of whom 65 experienced a hemorrhage (Table 1). The mean age of the cohort was 46.57 years (SD ± 18.39). When categorized according to bleeding status, the mean age in the bleeding group was 44.83 years (SD ± 15.50, range 8–78), while the non-bleeding group had a mean age of 48.01 years (SD ± 20.46, range 7–85, *p* = 0.30).

**Table 1:** Baseline characteristics of the reported patient cohort with cerebral cavernous malformations percentages refer to the total number of patients (*n* = 143).

Baseline characteristics	Number
Total patients	143
Total cavernomas	238
Patients with single cavernoma	118
Patients with multiple cavernomas	25
Familial (suspected or confirmed)	2 (1.4%)
Bleeding	65 (45.5%)
No bleeding	78 (54.5%)

62 patients (43.4%) were male, indicating a slight female predominance. In the patient group with cavernoma bleeding 34 from 65 were male (52.3%), against 28 from 78 without bleeding (35.9%, *p* = 0.6, two-sided Fisher's exact test).

Regarding cavernoma prevalence, 118 patients (82.5%) had a single cavernoma, while 25 patients (17.5%) had multiple cavernomas. Notably, only two patients were identified with suspected or confirmed familial cavernomatosis.

The mean follow-up time after diagnosis was 52.00 months (SD ± 60.54) in the bleeding group and 49.56 months (SD ± 57.44) in the non-bleeding group.

### 3.2 Symptoms

Among the 143 patients 35 (24.5%) were diagnosed with incidental cerebral cavernous malformations, while 106 patients presented with clinical symptoms (Table 2). Two additional patients were diagnosed during family screening due to suspected familial cavernomatosis. Of the 65 patients with hemorrhage, 57 were symptomatic. The remaining 8 were asymptomatic at the time of diagnosis—six were detected incidentally, and two were identified during familial screening.

**Table 2:** Frequency of symptoms in the cohort.

Symptoms	Overall cohort (*n* = 143)	Bleeding (*n* = 65)	Non-bleeding (*n* = 78)	*p*-value
Focal neurological deficit	25 (17.5%)	18 (27.7%)	7 (9%)	0.004
Epileptic seizure	23 (16.1%)	14 (21.5%)	9 (11.5%)	0.1157
Headache	24 (16.8%)	8 (12.3%)	16 (20.5%)	0.2616
Visual disturbances	7 (4.9%)	5 (7.7%)	2 (2.6%)	0.2454
Dizziness	27 (18.9%)	12 (18.5%)	15 (19.2%)	1.00
Total	106 (74.1%)	57 (87.7%)	49 (62.8)	0.001

The group of patients with detected hemorrhage and the group without bleeding are compared by two-sided Fishers exact test.

The most frequently reported symptoms were dizziness (18.9%) and focal neurological deficits (17.5%), followed by headache (16.8%) and epileptic seizures (16.1%). Visual disturbances were the least common, observed in only 4.9% of patients. Notably, clinical symptoms, and especially focal neurological deficits were significantly more frequent in the bleeding group compared to the non-bleeding group (Table 2). No significant differences between the two groups were observed for symptoms, such as headache, epileptic seizures, dizziness, and visual disturbances.

### 3.3 Lesion characteristics

The mean sagittal length of cavernomas in the overall cohort was 9.40 mm, with significant differences observed between the groups (11.08 mm vs. 8.00 mm, Table 3). Among the 143 patients, 51 lesions (35.7%) were located in arterial vascular border zones. The proportion of cavernomas in vascular border zones was 43.1% (28/65) in the bleeding group and 29.5% (23/78) in the non-bleeding group, without reaching significant difference between the two subsets (*p* = 0.115).

**Table 3:** Imaging characteristics of the index cavernoma (e.g., treated or observed, *t*-test).

Variable	Overall cohort (*n* = 143)	Bleeding (*n* = 65)	Non-bleeding (*n* = 78)	*p*-value
Length (sagittal) in mm	9.40 ± 6.74	11.08 ± 7.32	8.00 ± 5.91	0.006
Width (coronal) in mm	10.03 ± 6.95	12.24 ± 7.59	8.19 ± 5.81	0.001
Localization in vascular border zone (%)	51 (35.7%)	28 (43.1%)	23 (29.5%)	0.115

To provide a more detailed view, vascular border zones were further subclassified based on anatomical territories into ACA ACM, ACM ACP, ACP AB, and ACA ACP. Lesions in the ACM ACP region were significantly less frequently associated with bleeding (27.3%) compared to the other subtypes (*p* = 0.0007). No statistically significant differences were observed for the remaining sublocations (Table 4).

**Table 4:** Frequency of vascular border zone localizations among patients with and without hemorrhage.

Vascular border zone	Bleeding group	Non-bleeding group	*p*-value
ACA/ACM	11 (57.9%)	8 (34.8%)	0.779^b^
ACM/ACP	6 (27.3%)	16 (69.6%)	0.0007^b^
ACP/AB	6 (66.7%)	3 (13.0%)	0.487^b^
ACA/ACP	0	1 (4.3%)	0.451^c^
Total (*n* = 51)	28 (43.1%)	23 (29.5%)	0.115^a^

Groups are compared using two-sided Fisher's exact test.

^a^ Fisher's exact test comparing presence vs. absence of border zone localization.

^b^ Fisher's exact test comparing each subtype to all other border zone subtypes.

^c^ Not interpretable due to sample size of *n* = 1.

### 3.4 Risk factors

The univariate analysis revealed significant associations with bleeding for antithrombotic therapy (note the negative correlation, *p* = 0.012). Among the 143 patients, 24 (16.8%) received antithrombotic therapy. Within this subgroup, 13 patients (54.2%) were treated with cyclooxygenase inhibitors (e.g., acetylsalicylic acid), 6 (25.%) with DOACs NOACs (e.g., rivaroxaban, apixaban, dabigatran), 4 (16.7%) with vitamin K antagonists (e.g., phenprocoumon), and 1 patient (4.2%) with a P2Y12 inhibitor (e.g., clopidogrel). No patient received dual antiplatelet therapy.

Associations with bleeding events were also observed for increase in size during follow-up of the cavernous malformation (*p* = 0.002), and for the cavernomas shape (*p* = 0.001). In contrast, arterial hypertension, diabetes mellitus, gender and smoking habits were not found to have a significant association with cavernoma hemorrhage in the univariate model (Table 5).

**Table 5:** Univariate analysis of risk factors associated with bleeding.

Variable *n* (%)	Bleeding group (*n* = 65)	Non-bleeding (*n* = 78)	*p*-value
Cavernoma shape	34 cyclic (52.3%) 30 polycyclic (46.2%) 1 unknown (1.5%)	63 cyclic (80.8%) 14 polycyclic (17.9%) 1 unknown (1.3%)	0.001
Cavernoma growth (yes)	18 (27.7%)	6 (7.7%)	0.002
Antithrombotic medication (yes)	5 (7.7%)	19 (24.3%)	0.012
Gender (male)	34 (52.3%)	28 (35.9%)	0.062
Smoking (yes)	19 (29.2%)	15 (19.2%)	0.173
Hypertension (yes)	25 (38.5%)	34 (43.6%)	0.610
Diabetes mellitus (yes)	5 (7.7%)	4 (5.1%)	0.732

To further evaluate the independent effects of the variables on bleeding risk, a binary logistic regression model was constructed. Multicollinearity was assessed using variance inflation factors (VIF), and all variables included in the model exhibited VIF values below 2, indicating no significant multicollinearity among predictors (Table 6).

**Table 6:** Binary logistic regression analysis of risk factors for bleeding.

Variable	Odds ratio (OR)	95% confidence interval (CI)	*p*-value	Wald statistic
Antithrombotic medication	0.151	0.041-0.552	0.004	8.169
Male gender	2.114	1.047-4.269	0.037	4.362
Diabetes mellitus	4.962	0.841-29.274	0.077	3.129
Border zone	1.761	0.842–3.681	0.133	2.260
Epileptic seizure	1.360	0.573–3.226	0.486	0.486
Hypertension	1.228	0.561–2.690	0.608	0.264
Smoking	1.208	0.515–2.833	0.664	0.189

The use of antithrombotic medication remained significantly associated with a reduced risk of bleeding (OR: 0.151, 95% CI: 0.041–0.552, *p* = 0.004, Table 6). Male gender, which was not significant in the univariate analysis, was identified as an independent predictor of bleeding in the multivariate model (OR: 2.114, 95% CI: 1.047–4.269, *p* = 0.037). In contrast, the further factors, including diabetes mellitus, vascular border zone localization of the cavernoma, epileptic seizures, hypertension, and smoking habits, did not reveal significant associations to bleeding risk in the present cohort.

## 4 Discussion

Currently, the risk factors influencing hemorrhage in patients with cerebral cavernous malformations remain ambiguous, and clear therapeutic recommendations cannot be given. In this single-center retrospective analysis over a period of 23 years, 68 of the 143 patients experienced a hemorrhagic event. Additionally, we found that over 70% of patients presented with symptoms at the time of the initial diagnosis.

The central hypothesis of our study was that the anatomical localization of cavernomas within vascular border zones—also known as watershed areas—may be associated with an increased risk of hemorrhage. Border zones are regions located at the junctions of major cerebral arterial territories, where blood supply may be relatively compromised (16, 17). These territories are particularly vulnerable to slow capillary flow and relative hypoxia, which may lead to microthrombosis, venous congestion, and secondary rupture. This hemodynamic vulnerability is especially relevant in the context of CCMs, which are already prone to vascular instability. Thus, we hypothesized that lesions located in these territories might be more susceptible to bleeding.

In our cohort, 51 lesions (35.7%) were located in arterial vascular border zones. The proportion of cavernomas in vascular border zones was 43.1% (28/65) in the bleeding group and 29.5% (23/78) in the non-bleeding group, without reaching significant difference between the two subsets (*p* = 0.115). Notably, further sub-analysis revealed a significantly lower hemorrhage rate in the ACM/ACP subgroup (27.3%) compared to other border zone sublocations (*p* = 0.0007), potentially indicating heterogeneity within these territories. Although the global hypothesis of an increased bleeding risk in border zones was not statistically confirmed, these findings suggest that individual watershed areas may differ in their susceptibility to hemorrhage. This anatomical approach may still serve as a relevant framework for future investigations and risk stratification models. The negative result in our primary hypothesis does not diminish its clinical relevance, but rather calls for refined prospective studies incorporating microvascular imaging and hemodynamic mapping.

**Figure 2:**
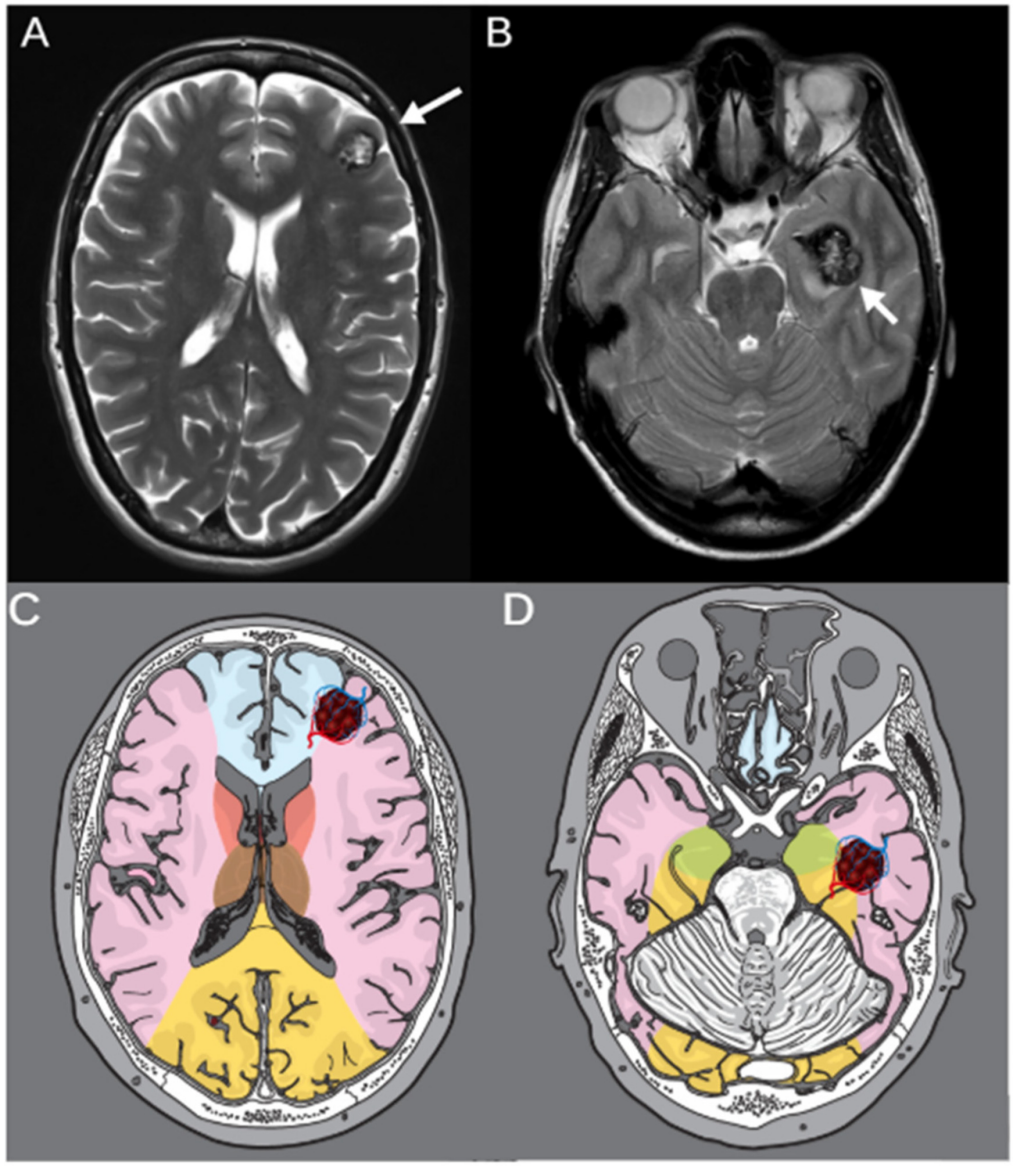
T2-weighted MRI of two representative cases with cerebral cavernomas located in vascular border zones. **(A)** Axial image of a 46-year-old female patient with a left frontal cavernoma in the ACA/ACM border zone. **(B)** Axial image of a 53-year-old patient with a cavernoma in the lefttemporal lobe, located in the ACM/ACP border zone. White arrows indicate the respective lesions. **(C,D)** Corresponding schematic illustrations of the same axial planes showing vascular territories and border zones. The vascular border zones are marked in red, and the respective cavernoma lesions are highlighted illustratively within the anatomical maps.

To illustrate the clinical relevance of vascular border zone localization, we included two representative cases with cavernomas located in different arterial watershed areas (Figure 2).

Our study supports the results of previous studies that had shown an association between a larger size of the cavernoma and hemorrhage (18, 19). The retrospective analysis also confirms that antithrombotic therapy in patients with cavernomas is inversely associated with bleeding, suggesting a protective effect against hemorrhagic events. This observation aligns with prior studies that support antithrombotic therapy's potential role in reducing bleeding risk in cerebral cavernous malformations (20, 21).

Antithrombotic agents are a broad category that includes both anticoagulants (e.g., vitamin K antagonist, DOACs) and antiplatelet medications (e.g., acetylsalicylic acid, clopidogrel), which act through distinct pathways (22). By preventing thrombus formation, these agents may help to avoid venous congestion and thus mitigate hemorrhagic events. Furthermore, certain antithrombotic agents improve cerebral blood flow by reducing microvascular occlusions (23), which may enhance the perfusion of surrounding brain tissue, potentially lowering ischemic injury and rupture risk. Some antithrombotic medications also promote endothelial stability (24), potentially reinforcing vascular integrity within CCMs. In addition, the anti-inflammatory properties of certain antithrombotic agents can stabilize these lesions (25), as inflammation has been linked to vascular fragility and increased rupture likelihood. Together, these mechanisms underscore an eventual therapeutic potential of antithrombotic therapy in managing bleeding risk in cavernoma patients.

Gender emerged as a significant risk factor for bleeding in our study, with male patients demonstrating a higher risk. This finding is consistent with published results, which identified male gender as a significant predictor of hemorrhage risk in patients with cavernous malformations (16). However, the literature on the influence of gender remains inconsistent. Some studies have suggested female sex as a risk factor for cavernoma-associated hemorrhage (4, 8, 17, 26), while others reported no clear gender predilection (27). These discrepancies may be attributed to differences in study design, population characteristics, or the inclusion of genetic and sporadic cases.

Smoking emerged as a potential risk factor for bleeding in our study, although it did not reach statistical significance. This observation aligns with the hypothesis that smoking-induced atherosclerosis exacerbates hemorheological changes and promotes blood flow disturbances within cavernomas (28). The literature presents conflicting evidence on this topic. Chen et al. found no significant association between nicotine abuse and hemorrhage risk in sporadic cavernomas (29), while Flemming et al. reported a significant link between tobacco use and increased lesion burden in familial cases, which could indirectly elevate the risk of bleeding (30). These contrasting findings underline the complex interplay between genetic predisposition, environmental factors, and cavernoma characteristics, underscoring the need for further research to clarify the role of smoking in cerebral cavernoma pathophysiology.

In our cohort, the size and the shape of the lesions are significantly correlated to hemorrhagic events (Tables 3, 5). Since hemorrhage by itself increases the size of the cavernoma and changes its shape, and since in this retrospective study 45% of the patients presented at the time of bleeding, this correlation is a symptom of the hemorrhage, rather than a prediction factor. On the other hand, after detection of an incidental cavernoma, increasing size and varying contours during follow-up MRIs should prompt alertness and re-discussion of the neurosurgical indication.

## 5 Limitations

The retrospective, single center design restricts causal conclusions, and potential bias may arise from reliance on medical records. In three emergency cases, diagnosis was based on CT imaging without MRI confirmation. Although these cases were reviewed and agreed upon in an interdisciplinary setting, the absence of MRI remains a diagnostic limitation.

The sample size limits statistical power, particularly in subgroup analyses, such as concerning the vascular border zone hemorrhage risk. Although we observed an absolute difference of over 10% between bleeding and non-bleeding groups regarding border zone localization, this did not reach statistical significance — likely due to insufficient statistical power. This limitation should be considered when interpreting the somewhat negative finding.

Furthermore, we did not differentiate detailed dosages of antithrombotic agents, which may have an impact on bleeding outcomes. Lastly, genetic factors were not analyzed, which could impact bleeding risk. Concerning analysis of brain imaging studies on shape and size of the cavernoma, these two items are strongly altered by the occurrence of the hemorrhage, thus making them associated to hemorrhage, but impeding to define them as true prognostic factors.

## 6 Conclusion

Our study confirms a reduced risk of bleeding in cavernoma patients treated with antithrombotic agents compared to those without such medication. Male gender was not significantly associated with bleeding in univariate analysis but emerged as an independent risk factor in the multivariate model. Irregular cavernoma shape, and changes in size during follow-up were also significantly associated with bleeding. Smoking habits were associated with a higher frequency of bleeding events, here, without reaching statistical significance.

While an overall impact of the localization of the lesion in an arterial watershed territory on the rate of hemorrhage could not be detected, we observed a higher bleeding rate in border zone cavernomas, together with a significantly lower bleeding frequency in lesions of the ACM ACP subgroup. The results presented here, thus remain equivocal regarding the clinical impact of cavernoma localization in vascular border zones.

## Data Availability

The raw data supporting the conclusions of this article will be made available by the authors, without undue reservation.
